# Effects of Comedy on the Health-Related Quality of Life of Cancer Survivors and Its Associations With the Big Five Personality Traits: A Post Hoc Analysis

**DOI:** 10.7759/cureus.57930

**Published:** 2024-04-09

**Authors:** Ryo Sakamoto

**Affiliations:** 1 Psychosomatic Medicine, Sakai City Medical Center, Sakai City, JPN

**Keywords:** oxidative stress index, personality traits, cancer survivor, quality of life (qol), comedy

## Abstract

Introduction: This study is a post hoc analysis of a single-arm trial to determine whether daily viewing of comedy videos for four weeks improves health-related quality of life (HRQOL) and oxidative stress in cancer survivors (UMIN-CTR 000044880). There are no reports of personality traits affecting HRQOL improvement. The purpose of this post hoc analysis was to identify associations with personality traits that may improve HRQOL.

Methodology: This analysis compared the baseline scores on the Ten-Item Personality Inventory-Japanese version (TIPI-J) for personality traits in Functional Assessment of Cancer Therapy-G (FACT-G) groups with improved or worsened scores. This grouping was based on the results of previous studies. In addition, the EuroQOL 5 dimension 3 level (EQ-5D-3L), Hospital Anxiety and Depression Scale (HADS) score, biological antioxidant potential (BAP), reactive oxygen metabolite-derived compounds, oxidative stress index, and potential antioxidant potential were assessed for each group. Items related to oxidative stress were tested using t-tests, while other items were tested using Friedman's analysis of variance.

Results: Forty-three participants completed the analysis (FACT-G improved [up group], *n *= 25; FACT-G decreased [down group], *n *= 18). No significant differences in the TIPI-J items existed between the two groups. Significant items for oxidative stress in the FACT-G up group were BAP (*P *= 0.04, Cohen's *d *= 0.32) and potential antioxidant capacity (*P *= 0.02, Cohen's *d *= 0.41). In the FACT-G down group, the significant item was potential antioxidant capacity (*P *= 0.03, Cohen's​​​​​​​ *d* = 0.46). The FACT-G up group had significant changes over time in the scores of the EuroQOL Visual Analog Scale (EQ-VAS; *χ*^2^ = 21.151 [df = 4]; *P* < 0.01), HADS-anxiety (χ^2^ = 24.579 [df = 4]; *P* < 0.01), and HADS-depression (χ^2^ = 29.068 [df = 4]; *P* < 0.01).

Conclusions: Our results suggested that cancer survivors’ personality traits did not influence the effects of viewing comedy. It has been suggested that the group with increased FACT-G may have had an improvement in the EQ-VAS, HADS, and potential antioxidant capacity independent of FACT-G.

## Introduction

In 2019, 23.6 million cases of cancer were expected to occur among 204 countries and regions [1]. Cancer is one of the most common diseases in Japan, with a cumulative cancer prevalence rate of 65% for men and 51% for women; most Japanese people experience some form of cancer [2]. With advances in cancer screening and treatment, survival rates are improving, and the number of cancer survivors is increasing [2]. By 2022, the United States is expected to have approximately 18.1 million cancer survivors, and this number is expected to continue to increase [3]. Cancer survivors experience various stressors after their diagnosis, treatment, and remission. Pain, neuropathy, fatigue, fear of recurrence, and reduced health-related quality of life (HRQOL) are a few examples [4-6]. Laughter is an alternative therapy that has been used.

Laughter therapy reduces depression, anxiety, and stress levels in patients with cancer [7]. However, few studies have demonstrated its effect on HRQOL. Laughter therapy has several classifications, but no clear definition has been established [8]. The authors of the current paper have conducted a previous intervention study [9] using ceremony video viewing as laughter therapy and reported its effects on HRQOL and oxidative stress. However, this study did not show any associations with background factors that may improve HRQOL such as personality traits [9]. There are no reports of personality traits affecting HRQOL improvement. Therefore, a post hoc analysis was conducted in this study to identify associations with personality traits that may improve HRQOL.

## Materials and methods

Study design and participants

**Figure 1:**
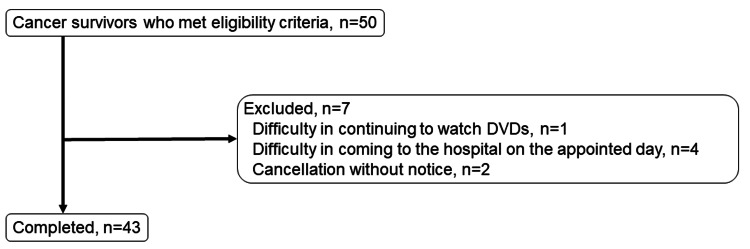
Flowchart of this study.

Data from an open-label, single-arm study in the University Medical Information Network (UMIN) Clinical Trials Registry (no. 000044880) were additionally analyzed. The study, which ran from April 2022 to March 2023, consisted of 15 minutes of viewing comedic videos daily for four weeks. This trial has been re-registered with the UMIN Clinical Trials Registry (no. 000061635). Details of the study design and main results have been reported previously [9]. Eligibility criteria were written informed consent, male or female sex, age 20 years or older, cancer diagnosis, and cancer status cured or stable. The exclusion criteria were patients undergoing treatment for cancer recurrence or metastasis, patients whose condition was unstable, patients taking contraceptive pills, and patients in the terminal stage of cancer. This study was conducted per the ethical principles of the Declaration of Helsinki and in compliance with the International Conference on Harmonization Good Clinical Practice guidelines and applicable regulatory requirements. All patients provided written informed consent before enrollment. A flowchart of this study is shown in Figure 1.

The primary endpoint of this post hoc analysis was a comparison of the results of the Ten-Item Personality Inventory-Japanese version (TIPI-J) items (i.e., Conscientiousness, Agreeableness, Neuroticism, Openness to Experience, and Extraversion) [10] between the two groups: (1) the group with an increase in the Functional Assessment of Cancer Therapy-General (FACT-G) score [11] and (2) the group with a decrease in the FACT-G score. This grouping was based on the results of previous studies [9].

The secondary endpoints were age, number of days since the cancer diagnosis, whether they found comedy funny; stress before and after the COVID-19 epidemic; and their preference for the comedy DVDs used in the study, rated on a 0- to 10-point Likert scale; and the scores of the EuroQOL 5-dimension 3-level (EQ-5D-3L) [12], EuroQOL Visual Analog Scale (EQ-VAS) [12], and Hospital Anxiety and Depression Scale (HADS) [13]. EQ-5D-3L is used to comprehensively assess a patient's HRQOL and includes five questions addressing the level of mobility, personal care, usual activities, pain or discomfort, and anxiety or depression. EQ-VAS rates participants' self-reported health status on a scale from 0 (poor health) to 100 (best health). HADS is used to measure psychiatric symptoms (anxiety and depression) in patients with physical illnesses. HADS has 14 items, seven for depression and seven for anxiety. Based on the biological antioxidant potential (BAP) is an indicator of reducing power (antioxidant power), which is the degree to which iron oxide can be reduced, and this is measured via a redox reaction [14]. The reactive oxygen metabolite-derived compounds (d-ROMs) test is a method to assess the level of oxidative stress in vivo by measuring hydroperoxides produced by free radicals and reactive oxygen species in vivo using a colorimetric reaction [15]. Oxidative Stress Index (OSI) is calculated by multiplying the ratio of d-ROM/BAP by 100. Combining ROM and BAP data rather than treating them separately provides more accurate information about the level of oxidative stress [16]. Potential antioxidant capacity was calculated using the equation: BAP/d-ROMs. The level of oxidative stress was evaluated according to the balance between oxidative level and antioxidant capacity, and the ratio of oxidative level to antioxidant capacity is often used as an indicator [17]. Oxidative stress scores [BAP], d-ROMs, OSI, and potential antioxidant capacity were compared between the first and last day of the study in the two groups.

Assessments

Participants were asked to complete demographic information on the first day of the study and to complete a questionnaire on the last day. On the first day, participants provided the following items: age, sex, marital status, occupation, cancer tumor, number of days since cancer diagnosis, presence of noncancer diseases, and whether they were in the habit of watching comedic videos. In addition, they reported whether they liked comedy on a Likert scale of (0 [does not like at all] to 10 [does like extremely]), and they completed the TIPI-J. The TIPI-J was used to assess a patient’s big five personality traits. It consists of 10 questions about the patient’s extraversion, cooperation, industriousness, neuroticism, and openness. The reliability and validity of the instrument have been validated previously [10].

Each scale has high internal consistency: Extraversion (α = 0.92), Agreeableness (α = 0.85), Conscientiousness (α = 0.82), Emotional Stability (α = 0.91), and Openness to Experience (α = 0.86) [10]. The following items were completed on the last day of the study: stress in daily life before and after the COVID-19 pandemic, reported on a Likert scale of 0 (no stress at all) to 10 highest stress); whether the comedy DVDs borrowed in this study met their preferences, reported on a Likert scale of 0 (no preference at all) to 10 (highest preference); and whether a major life event occurred during the study period, reported with *yes*/*no* (e.g., death in the family, divorce, separation, new illness, etc.). The ED-5D-3L, EQ-VAS, and HADS questionnaires were administered at baseline (T1), one week later (T2), two weeks later (T3), three weeks later (T4), and four weeks later (T5).

Statistical analyses

Each item assessed on the first or last day of the study in the FACT-G increased score (FACT-G up) group and FACT-G decreased score (FACT-G down) group was compared by using t-tests. Differences between the ED-5Q-3L, EQ-VAS, and HADS scores at different time points (i.e., T1, T2, T3, T4, and T5) in the two groups were also tested by using nonparametric Friedman analysis of variance. Statistical analysis was conducted using R software, version 4.3.2 (R Foundation for Statistical Computing, Vienna, Austria). Statistical significance was set at *P* < 0.05.

## Results

The study included 50 participants (10 men and 40 women). However, three men and four women dropped out of the study before the four-week follow-up because of scheduling conflicts or other reasons. Thus, the final sample for this study consisted of 43 individuals: 25 in the FACT-G up group and 18 in the FACT-G down group. Table 1 summarizes the baseline characteristics of the two groups.

**Table 1:** Characteristics of our study population (n = 43). TIPI-J, Ten-Item Personality Inventory-Japanese; FACT-G, Functional Assessment of Cancer Therapy-G

Characteristics	Increased FACT-G（*n *= 25）	Decreased FACT-G (*n *= 18)	*P*-value
Age (years)			
Mean (SD)	58.24 (12.11)	59.38 (8.29)	0.71
Sex, *n* (%)			
Male	5 (20)	2 (11)	
Female	20 (80)	16 (89)	
Married, *n* (%)	19 (76)	12 (67)	
Job, employed, *n* (%)	20 (80)	11 (61)	
Cancer type (some with two onsets), *n* (%)			
Breast	15 (60)	5 (28)	
Colorectal	3 (12)	5 (28)	
Uterine	1 (4)	6 (33)	
Lung	2 (8)	1 (6)	
Other	6 (24)	2 (11)	
Months after cancer onset, mean (SD)	8.36 (7.64)	7.28 (4.29)	0.48
Hospital visit for noncancer, *n* (%)	13 (52)	10 (56)	
The habit of watching comedy, *n* (%)	12 (48)	9 (50)	
Major life events during this study, *n* (%)	5 (20)	4 (22)	
TIPI-J, *n* (%)			
Openness to experience	4.32 (1.05)	4.44 (1.39)	0.75
Extraversion	4.62 (1.35)	5.11 (1.20)	0.21
Agreeableness	5.12 (1.04)	5.33 (0.89)	0.47
Conscientiousness	3.82 (1.29)	3.77 (1.41)	0.92
Neuroticism	4.12 (1.27)	4.27 (1.44)	0.71
COVID-19 before epidemic	4.44 (2.06)	3.77 (2.28)	0.33
COVID-19 post epidemic	6.28 (2.11)	5.72 (2.19)	0.41
Can you find comedy funny?	6.24 (1.80)	7.29 (2.11)	0.03
DVD preferences used in this study	5.50 (3.28)	5.83 (2.79)	0.71

More than 80% of the participants in both groups were female, and more than 50% had comorbidities other than cancer, most commonly breast cancer in the FACT-G up score group and uterine cancer in the FACT-G down group. Viewing comedy was a habit among approximately 50% of both groups. Approximately 20% experienced a major life event during the study period.

TIPI-J

The comparison of the means of the TIPI-J scores (items: Conscientiousness, Agreeableness, Neuroticism, Openness to Experience, and Extraversion) is presented in Table 1. Paired t-tests revealed no significant differences in the scores for Openness to Experience (*P *= 0.75), Extraversion (*P* = 0.21), Agreeableness (*P* = 0.47), Conscientiousness (*P* = 0.92), and Neuroticism (*P* = 0.71).

Other questions include age, months after cancer onset, *Can you find comedy funny?*, COVID-19 before the epidemic, COVID-19 after the epidemic, and the *DVD preferences used in this study*.

A comparison of the means of the six items is shown in Table 1. Paired t-tests revealed significant differences in the results for *Do you think comedy is funny?* (*P* < 0.03, Cohen's *d* = 0.54), thereby indicating moderate effects. The results for other items were not significantly different.

EQ-5D-3L, EQ-VAS, and HADS

The scores of the EQ-5D-3L, EQ-VAS, and HADS questionnaires at T1, T2, T3, T4, and T5 were compared. In the FACT-G up group, significant changes occurred over time in the scores of the EQ-VAS (*χ*^2^= 21.151 [df = 4], *P* < 0.01), HADS-A (χ^2^ = 24.579 [df = 4], *P* < 0.01], and HADS-D (*χ*^2^ = 29.068 [df = 4], *P* < 0.01). Post hoc tests revealed that these three items were significantly different at baseline, three weeks (T4), and four weeks (T5). Table 2 presents the details of the other results. In the FACT-G down group, only the HADS-A score changed significantly over time (*χ*^2^ = 11.06 [df = 4]; *P* = 0.02). However, as shown in Table 3, the post hoc tests revealed no significant differences.

**Table 2:** EQ-5D-3L, EQ-VAS, and HADS score intervals at baseline and weekly intervals in the FACT-G up group. Significance of the mean difference using the Holm adjustment for multiple comparisons. A *P*-value of 0.05 or less was considered significant. ^*^P-value < 0.05. EQ-5D-3L, EuroQOL 5 dimension 3 level; EQ-VAS, EuroQOL Visual Analog Scale; HADS, Hospital Anxiety and Depression Scale; FACT-G, Functional Assessment of Cancer Therapy-General

	T1	T2	T3	T4	T5	T1 to T2	T1 to T3	T1 to T4	T1 to T5	T2 to T3	T2 to T4	T2 to T5	T3 to T4	T3 to T5	T4 to T5
	Mean (SD)					Sig									
EQ-5D-3L	0.86 (0.12)	0.85 (0.13)	0.89 (0.13)	0.81 (0.21)	0.90 (0.12)	*P *= 1.00	*P *= 1.00	*P *= 1.00	*P *= 1.00	*P *= 1.00	*P *= 1.00	*P *= 0.66	*P *= 1.00	*P *= 1.00	*P *= 1.00
EQ-VAS	72 (15.87)	85 (20.94)	83.31 (21.37)	81.16 (12.45)	82.36 (12.60)	*P *= 0.14	*P *< 0.01*	*P *< 0.01*	*P *< 0.01*	*P *= 0.92	*P *= 1.00	*P *= 0.92	*P *= 1.00	*P *= 1.00	*P *= 1.00
HADS-A	6.88 (4.59)	4.48 (3.67)	3.72 (3.30)	4.16 (3.15)	3.56 (3.08)	*P *= 0.01*	*P *= 0.06	*P *= 0.01*	*P *< 0.01*	*P *= 1.00	*P *= 1.00	*P *= 0.46	*P *= 1.00	*P *= 0.54	*P *= 0.88
HADS-D	6.36 (3.34)	5.76 (3.53)	5.81 (3.66)	4.56 (2.66)	3.52 (3.53)	*P *= 0.38	*P *= 0.27	*P *= 0.02*	*P *< 0.01*	*P *= 0.75	*P *= 0.10	*P *< 0.01*	*P *= 0.19	*P *= 0.02*	*P *= 0.07

**Table 3:** EQ-5D-3L, EQ-VAS, and HADS score intervals at baseline and weekly intervals in the FACT-G down group. Significance of the mean difference using the Holm adjustment for multiple comparisons. A *P*-value of 0.05 or less was considered significant. EQ-5D-3L, EuroQOL 5 dimension 3 level; EQ-VAS, EuroQOL Visual Analog Scale; HADS, Hospital Anxiety and Depression Scale; FACT-G, Functional Assessment of Cancer Therapy-General

	T1	T2	T3	T4	T5	T1 to T2	T1 to T3	T1 to T4	T1 to T5	T2 to T3	T2 to T4	T2 to T5	T3 to T4	T3 to T5	T4 to T5
	Mean (SD)					Sig									
EQ-5D-3L	0.84 (0.20)	0.89 (0.19)	0.92 (0.11)	0.96 (0.24)	0.90 (0.11)	*P *= 0.41	*P *= 0.41	*P *= 0.47	*P *= 1.00	*P *= 1.00	*P *= 1.00	*P *= 1.00	*P *= 1.00	*P *= 1.00	*P *= 0.73
EQ-VAS	74.16 (16.91)	77.22 (13.19)	82.75 (23.16)	79.55 (12.74)	80.05 (11.94)	*P *= 1.00	*P *= 0.74	*P *= 1.00	*P *= 1.00	*P *= 0.07	*P *= 1.00	*P *= 1.00	*P *= 0.07	*P *= 1.00	*P *= 1.00
HADS-A	5.66 (3.78)	4.94 (3.45)	4.83 (3.77)	3.72 (3.02)	4.77 (3.90)	*P *= 0.74	*P *= 0.53	*P *= 0.10	*P *= 0.74	*P *= 1.00	*P *= 0.23	*P *= 1.00	*P *= 0.47	*P *= 1.00	*P *= 0.24
HADS-D	5.22 (2.57)	5.91 (2.99)	5.61 (3.43)	4.27 (3.23)	4.61 (3.34)	*P *= 1.00	*P *= 1.00	*P *= 0.91	*P *= 1.00	*P *= 1.00	*P *= 0.65	*P *= 1.00	*P *= 0.29	*P *= 0.91	*P *= 1.00

BAP, d-ROMs, OSI, and potential antioxidant capacity

A comparison of the means of all items in the biological analyses is shown in Table 4. The t-test results showed a significant difference between T1 and T5 for BAP (*P* = 0.04, Cohen's *d* = 0.32) and potential antioxidants (*P* = 0.02, Cohen's *d* = 0.41) for the FACT-G up group. The effect size was small to moderate. The d-ROM (*P* = 0.46) and OSI (*P* = 0.13) were not significantly different. For the FACT-G down group, there was a significant difference between T1 and T5 for potential antioxidants (*P* = 0.03, Cohen's *d* = 0.46). The effect sizes ranged from small to moderate. The d-ROMs (*P* = 0.84), BAP (*P* = 0.05), and OSI (*P* = 0.06) were not significantly different.

**Table 4:** Two groups' levels of d-ROM, BAP, and OSI before and after the study. ^*^P-value < 0.05. d-ROM, reactive oxygen metabolite-derived compound; BAP, biological antioxidant potential; OSI, Oxidative Stress Index; FACT-G, Functional Assessment of Cancer Therapy-General

	Increased FACT-G (*n *= 25)			Decreased FACT-G down (*n *= 18)		
Markers	Pre-mean (SD)	Post-mean (SD)	*P*-value	Pre-mean (SD)	Post-mean (SD)	*P*-value
d-ROMs (CARR unit)	347.88 (85.38)	340.04 (108.65)	0.46	314.83 (66.88)	318.94 (50.79)	0.84
BAP (μmol/L)	1743.88 (603.23)	1911.03 (412.87)	0.04*	1549.82 (791.88)	1862.8 (436.74)	0.05
OSI	3.37 (5.92)	1.68 (0.67)	0.13	2.92 (3.26)	1.67 (0.83)	0.06
Potential antioxidant capacity	5.23 (2.01)	6.06 (2.02)	0.02*	5.13 (2.37)	6.08 (1.71)	0.03*

## Discussion

The purpose of this study was to complement the analysis of a previous study [9] that investigated the effectiveness of four weeks of viewing comedy on HRQOL and oxidative stress in cancer survivors. The participants were divided into two groups - the FACT-G up group and the FACT-G down group - to determine whether personality traits or other characteristics influenced the effects of comedy. We also evaluated changes in oxidative stress, HRQOL, and psychiatric symptoms over time in each group.

An important finding of this study was that no relationship existed between personality traits and the effect of comedy. For example, family therapy may be influenced by conscientiousness and individual therapy may be influenced by extraversion for better treatment effects [18]. Neurotic tendencies are associated with a significantly lower treatment response [19]. Thus, while personality traits influence treatment efficacy, the effects of comedy may not be influenced by personality traits. Therefore, we consider this factor to be a strong advantage in that it can be easily introduced in a variety of situations.

Another important aspect is that the effect of comedy is more likely to be observed among people who do not normally enjoy it. Reports indicate that comedic videos, written literature, movies, etc., are beneficial for immunity, analgesia, and stress reduction; however, the results are inconsistent [20]. However, watching comedic videos positively affects pain by increasing positive emotions [21]. People are programmed to adapt to subjective emotions [22]. However, people who normally enjoy comedy may be less likely to experience positive emotions because of habituation, thereby making comedy less effective.

These results are interesting, suggesting that potential antioxidant capacity may be improved regardless of whether the FACT-G score is increased or decreased. Decreased HRQOL associated with emotional disorders is also associated with worsened oxidative stress [23]. The reason for the improvement in the FACT-G score in this study, regardless of whether the score improved or worsened, may be that the four-week daily regimen was effective. Improvements in FACT-G may also be useful for improving depression and anxiety scores. Higher levels of pessimism have been reported to increase the risk of depression and anxiety and decrease HRQOL [24]. The level of pessimism, although not investigated in this study, may have affected the effectiveness of comedy.

The limitations of our study include the post hoc nature of the analysis and the small sample size of the subgroups. These factors limit the interpretation of the results. Further confirmatory analyses in larger patient populations are required to validate these results. Finally, no restrictions were placed on new health-related activities such as the consumption of antioxidant foods or initiation of exercise during the study period. Therefore, engaging in these activities may have affected the results of some participants.

This study is a relatively simple intervention that can be practiced, although an interest in comedy will increase the likelihood that they will continue to do so. There are still few reports on the relationship between comedy and medical care, and we believe that this study could help accumulate evidence for such studies. Future comparative studies should be conducted to improve the quality of the results of this study.

## Conclusions

The effects of comedy on cancer survivors may not be influenced by personality traits. It was suggested that the group with increased FACT-G may have improved EQ-VAS, HADS, and potential antioxidant capacity regardless of FACT-G. High-quality research based on this study is needed, but it may be an option for cancer survivors as a non-drug intervention tool that is not influenced by personality traits. Comedy is safe, convenient, and inexpensive, making it an extremely valuable intervention for cancer survivors.
